# Superomedial Scapula Angle Osteochondroma with Winging in a Young Female Patient—Case Report and Literature Review

**DOI:** 10.3390/jcm12155106

**Published:** 2023-08-03

**Authors:** Cosmin Faur, Ahmed Abu-Awwad, Jenel-Marian Patrascu, Simona-Alina Abu-Awwad, Cristina Tudoran

**Affiliations:** 1 Department XV—Discipline of Orthopedics—Traumatology, “Victor Babes” University of Medicine and Pharmacy, Eftimie Murgu Square No. 2, 300041 Timisoara, Romania; faur17@gmail.com (C.F.); jenelmarianp@yahoo.com (J.-M.P.J.); 2 “Pius Brinzeu” Emergency Clinical County Hospital, Bld Liviu Rebreanu No. 156, 300723 Timisoara, Romania; alina.abuawwad@umft.ro (S.-A.A.-A.); cristina13.tudoran@gmail.com (C.T.); 3 Research Center University Professor Doctor Teodor Șora, Victor Babes University of Medicine and Pharmacy, Eftimie Murgu Square No. 2, 300041 Timisoara, Romania; 4 Department XII—Discipline of Obstetrics and Gynecology, Victor Babes University of Medicine and Pharmacy, Eftimie Murgu Square No. 2, 300041 Timisoara, Romania; 5 Discipline of Cardiology, Victor Babes University of Medicine and Pharmacy, Eftimie Murgu Square No. 2, 300041 Timisoara, Romania

**Keywords:** superomedial scapula angle, osteochondroma, scapula winging, benign bone tumors, surgical excision

## Abstract

(1) Background: Osteochondromas are the most common benign bone tumors, primarily found in long bones, while scapular osteochondromas are rare and account for less than 1% of all osteochondromas. (2) Methods: We present a case of a young female patient with a unique presentation of scapular osteochondroma. The patient exhibited superomedial scapula angle osteochondroma with winging, a rare manifestation of scapular osteochondroma. The patient had a slow-growing mass on the left scapula for several years. Physical examination revealed a visible deformity with significant winging of the scapula. Imaging studies demonstrated a large osteochondroma arising from the superomedial angle of the left scapula, with a bony stalk. (3) Results: Surgical excision was performed, and histopathological analysis confirmed the diagnosis of osteochondroma. Following the surgery, the patient experienced a significant improvement in scapular winging. A comprehensive literature review revealed only a limited number of reported cases of scapular osteochondroma with winging, underscoring the significance of this case report as a valuable addition to the existing literature. The diagnosis of scapular osteochondroma should be considered in the differential diagnosis of patients presenting with a scapular mass, particularly when associated with winging. Surgical excision is the recommended treatment, and complete excision is crucial to prevent recurrence. (4) Conclusions: This case report highlights a rare presentation of scapular osteochondroma with winging and emphasizes the importance of considering this diagnosis in patients with scapular masses accompanied by winging. The successful surgical intervention in our case resulted in significant improvement. Clinicians should be aware of this entity and consider prompt surgical intervention for complete excision, ensuring optimal patient outcomes and preventing recurrence. Further research and additional case reports are necessary to enhance our understanding of scapular osteochondroma and its varied clinical presentations. Furthermore, comprehensive studies involving larger patient cohorts are necessary to explore the full spectrum of clinical presentations of scapular osteochondromas. By documenting and analyzing a wider range of cases, including variations in tumor location, size, and associated symptoms, researchers can identify patterns and establish more accurate diagnostic criteria. This will facilitate early detection and appropriate management of scapular osteochondromas, ultimately improving patient outcomes.

## 1. Introduction

Scapular winging is a distinct condition characterized by the abnormal protrusion of the scapula from the back, giving it a noticeable “wing-like” appearance [1]. This positioning anomaly occurs as a result of the weakening or paralysis of the muscles responsible for connecting the scapula to the thorax, leading to the scapula being pulled away from its normal anatomical position. While nerve injuries, particularly those affecting the long thoracic nerve or the spinal accessory nerve, are the most common causes of scapular winging, bony abnormalities such as osteochondromas can also contribute to the development of this condition [2].

Osteochondromas are benign bone tumors that have the potential to develop in any bone. However, they are most frequently encountered in the long bones of the arms and legs. Osteochondromas originating in the scapula are considered rare, and those specifically located in the superomedial angle of the scapula are even rarer. These superomedial scapular angle osteochondromas present unique challenges in terms of diagnosis and management. Understanding the clinical presentation, diagnostic approaches, treatment options, and outcomes associated with this rare manifestation is crucial for providing appropriate care to affected patients [3].

Superomedial scapular angle osteochondroma with winging is a rare condition, with an estimated prevalence of 0.003% to 0.02%. This means that it is estimated that there are only 3 to 20 people in every 100,000 people who have this condition. The condition is more common in females than males, with a female-to-male ratio of 2:1 [2,3,4].

This article aims to present a noteworthy case of superomedial scapular angle osteochondroma associated with scapular winging in a young female patient. By analyzing this case, we aim to shed light on the clinical characteristics and management strategies specific to this rare condition. Furthermore, a comprehensive review of the existing literature on scapular osteochondromas and associated winging will be conducted to further enhance our understanding of this unique manifestation.

Through an in-depth examination of the presented case and a thorough review of the literature, we hope to contribute valuable insights into the clinical features, diagnostic modalities, treatment options, and prognostic considerations associated with superomedial scapular angle osteochondroma with winging. This information will not only enhance our knowledge of this rare condition but also assist healthcare professionals in making informed decisions regarding diagnosis and management.

Scapular winging caused by osteochondromas is a rare manifestation that can significantly impact a patient’s quality of life. While nerve injuries are the most common cause of scapular winging, bony abnormalities such as osteochondromas can also contribute to its development. The occurrence of osteochondromas in the scapula, particularly in the superomedial angle, is considered rare [4]. This article presents a unique case of superomedial scapular angle osteochondroma associated with scapular winging in a young female patient and provides a comprehensive review of the literature to enhance our understanding of this rare occurrence. By examining the clinical features, diagnostic approaches, treatment options, and outcomes associated with this condition, we aim to contribute to the existing body of knowledge and promote better management of patients with this unique presentation. The presented case involves an individual with scapular winging caused by a superomedial scapular angle osteochondroma. The patient exhibited a slow-growing mass on the left scapula that had been present for several years. Physical examination revealed a visible deformity with significant winging of the scapula. Imaging studies, including radiographs or other modalities, confirmed the presence of a large osteochondroma arising from the superomedial angle of the left scapula, with a bony stalk.

The rarity of this case highlights the need for a comprehensive literature review to identify and analyze previously reported instances of scapular osteochondromas with associated winging. By consolidating the available information, healthcare professionals can gain valuable insights into the diagnosis, management, and treatment outcomes of this unique presentation. This case report not only adds to the existing literature but also emphasizes the importance of considering scapular osteochondromas in the differential diagnosis of patients presenting with scapular masses, particularly when scapular winging is observed.

Osteochondromas, benign osseous tumors arising from growth plates, predominantly manifest in long bones, and when affecting the scapula, may induce complications such as winging given their proximity to nerves and soft tissues. Radiologically, they are characterized via:

X-ray:

- Exostosis: Evident as a distinct bony projection from the scapular cortical bone, typically orienting away from the closest joint;
- Bone continuity: Osteochondroma cortical bone is in seamless continuity with the host scapular bone, as is the marrow space;
- Skeletal alteration: Osteochondroma may precipitate visible scapular deformity.

MRI:

- Cartilaginous cap: Osteochondromas characteristically bear a cartilaginous cap, observable on MRI as a high-intensity signal on T2-weighted images; a cap exceeding 1–2 cm thickness in adults could indicate a rare malignant transformation;
- Bone marrow continuity: MRI echoes X-ray findings of osteochondroma and host marrow continuity;
- Soft tissue implication: MRI can offer intricate details on the osteochondroma’s influence on surrounding soft tissues, inclusive of muscles, nerves, and vessels.

These are typical findings; however, variations exist, and diagnosis should align with clinical history and presentation.

In conclusions, scapular winging is a distinct condition characterized by the protrusion of the scapula, primarily caused by muscle weakness or paralysis. While nerve injuries are the most common etiology, bony abnormalities such as osteochondromas can also contribute to this condition. Superomedial scapular angle osteochondromas are rare occurrences, further underscoring the significance of this case report. By reviewing the literature and sharing this unique case, we contribute to the understanding and awareness of scapular osteochondromas with associated winging.

## 2. Materials and Methods

### 2.1. Case Report

**Figure 1:**
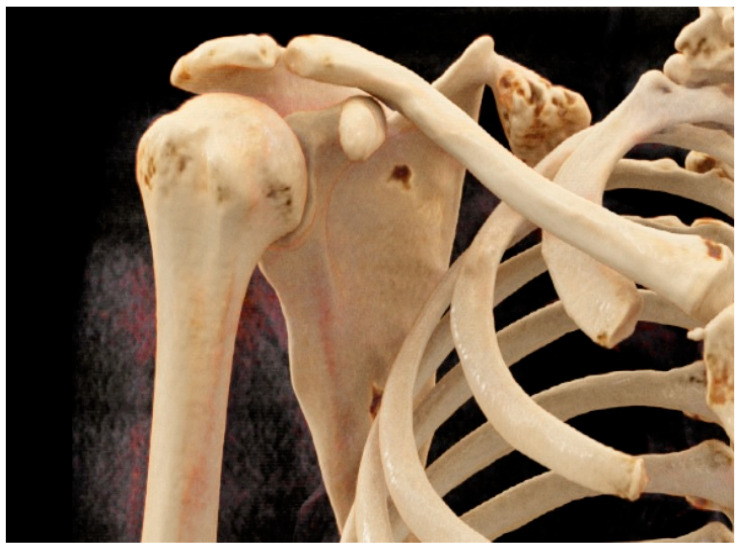
The bony mass in the superomedial angle of the left scapula consistent with an osteochondroma (computed tomography scan).

A 24-year-old female presented to our clinic with a six-month history of pain and swelling in her right shoulder. The patient presented with a protrusion of the left scapula, emanating from the dorsal region, leading to significant discomfort. On physical examination, we noted a prominent left scapula that was winging from the back. There was also a palpable, firm mass in the superomedial angle of the left scapula. Imaging revealed a bony mass in the superomedial angle of the left scapula consistent with an osteochondroma (Figure 1).

**Figure 2:**
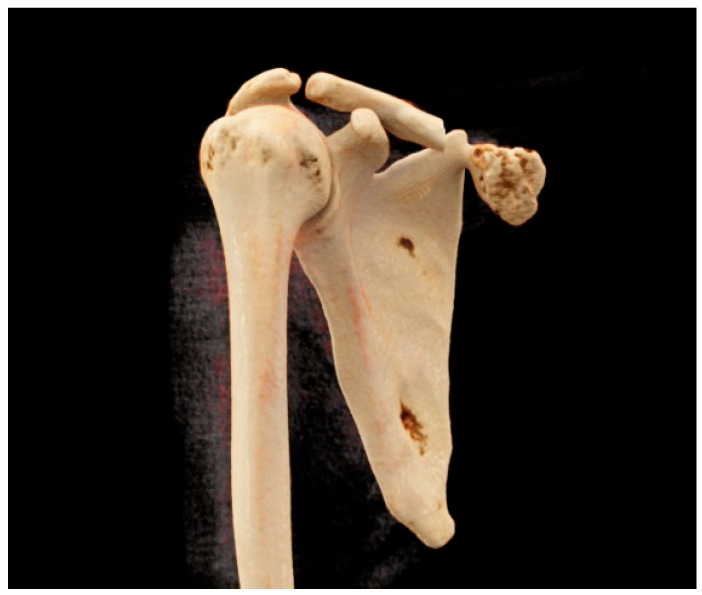
Erosion of the scapular bone (computed tomography scan).

CT showed that the mass was attached to the scapula and had caused erosion of the scapular bone (Figure 2).

The patient underwent surgical excision of the osteochondroma, and her symptoms improved postoperatively. Follow-up radiographs at 1, 3, 6, and 12 months showed no evidence of recurrence. At present, the patient’s follow-up is conducted every 6 months, accompanied by regular radiological investigations.

The management and treatment of scapular winging with osteochondromas depend on the severity of the symptoms and the size and location of the osteochondroma. In asymptomatic cases, observation may be appropriate, with regular monitoring to detect any changes in the size or symptoms of the osteochondroma.

However, in cases where the osteochondroma is causing significant pain, weakness, or functional limitations, surgical excision is the treatment of choice. The goal of surgery is to remove the osteochondroma and any associated bone or soft tissue that may be contributing to the scapular winging. In some cases, the scapula may need to be stabilized or repositioned during surgery to prevent recurrence of the scapular winging.

There are several surgical approaches to the excision of scapular osteochondromas, including open or arthroscopic approaches. The choice of approach depends on the size and location of the osteochondroma, as well as the surgeon’s preference and expertise.

### 2.2. Technique

**Figure 3:**
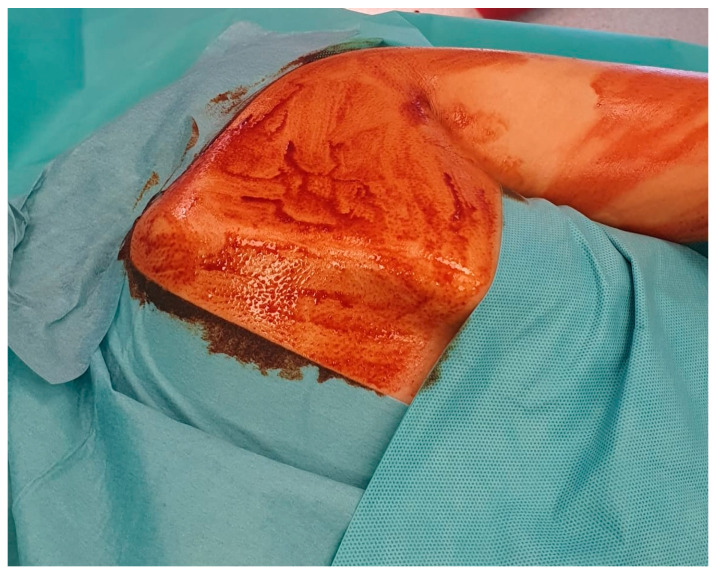
Surgical skin incision.

A 5 cm longitudinal incision, 0.5 cm medial to the medial border of the right scapula, centered on the superomedial angle. Following skin incision and dissection of the fat tissue, a split of the rhomboid fibers was performed, revealing the base of the tumoral mass (Figure 3).

Carefully, peritumoral dissection was continued up to the tip of the scapula, palpating the contour of the second rib which was in contact with the tumor.

Resection of the base of the tumor was performed with an oscillating saw followed by detachment and removal of the osteochondroma.

**Figure 4:**
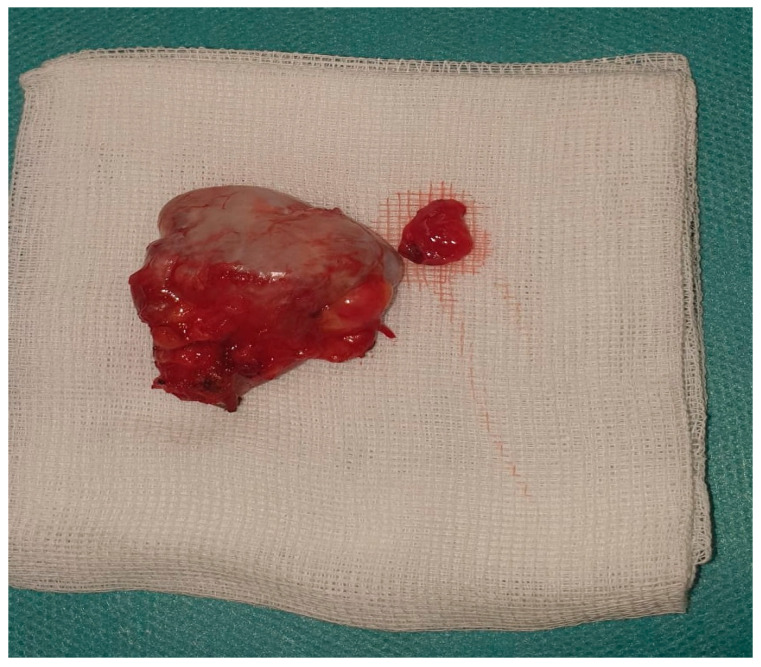
Mass tumor.

**Figure 5:**
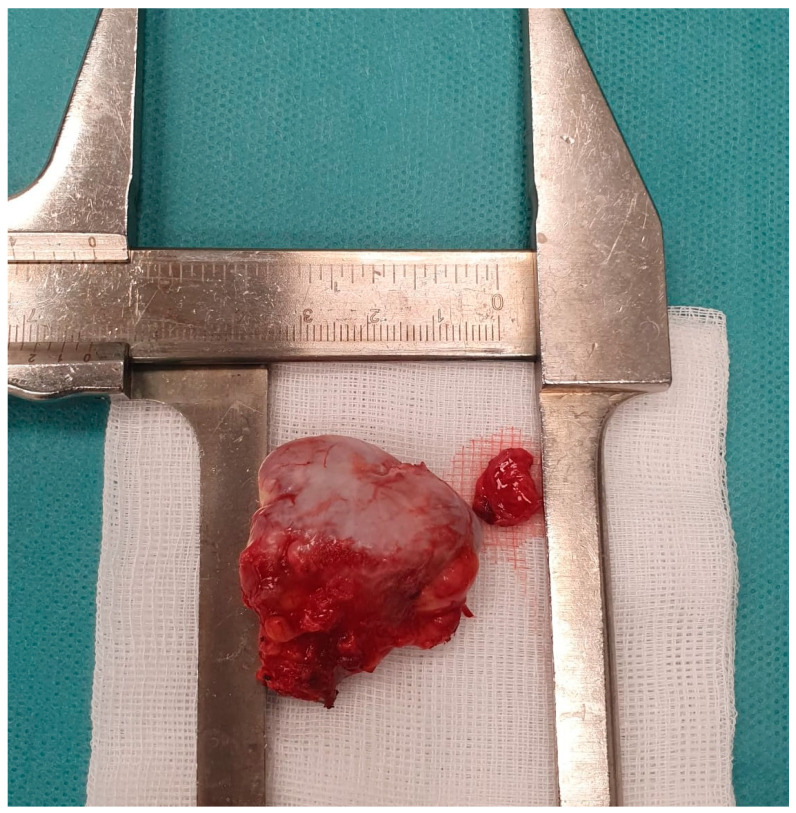
Measurement of the mass tumor.

Carefully, bleeding control was assured together with excision of the bursal peritumoral tissue (Figure 4 and Figure 5).

Closure with interrupted sutures of the subcutaneous tissue and intradermic suture with performed at the end of the procedure.

The sutures were removed at 14 days postop.

After surgical excision, patients may need to undergo physical therapy to restore strength and range of motion to the shoulder and scapula. They may also need to wear a brace or sling for several weeks to support the shoulder and allow for proper healing.

**Figure 6:**
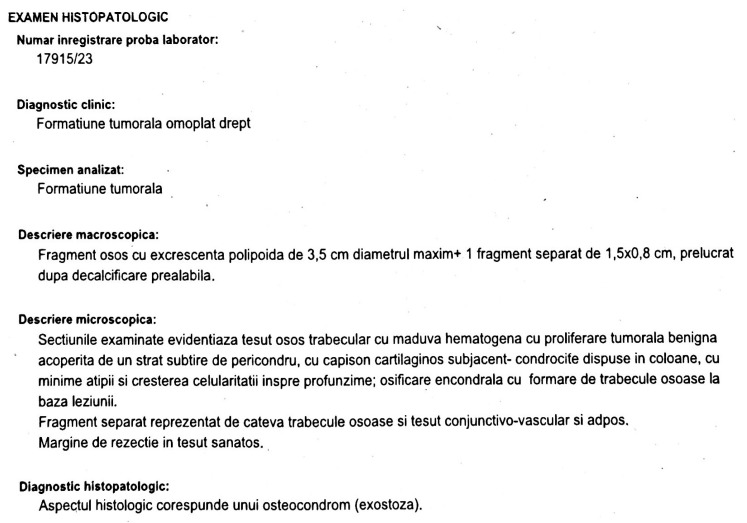
Histopathological examination performed on the surgically removed tissue sample.

The histopathological examination performed on the surgically removed tissue sample shows us that the existing tumor mass is an osteochondroma (Figure 6).

Histopathological Examination Report

Laboratory sample registration number: 17915/23.

Specimen analyzed: Tumor formation in the right shoulder blade.

Macroscopic Description:

The specimen consists of a bone fragment with a polypoid excrescence measuring 3.5 cm in maximum diameter, along with a separate fragment measuring 1.5 × 0.8 cm^2^. The specimens were processed after prior decalcification.

Microscopic Description:

Microscopic examination of the sections reveals trabecular bone tissue with hematogenous marrow. The bone tissue shows benign tumor proliferation, which is covered by a thin layer of perichondrium. A subjacent cartilaginous cap is present, composed of chondrocytes arranged in columns. These chondrocytes exhibit minimal atypia and increased cellularity towards the depth of the lesion. Endochondral ossification is observed, with the formation of bone trabeculae at the base of the lesion.

The separate fragment consists of a few bone trabeculae along with conjunctive-vascular and adipose tissue. The resection margin shows healthy tissue.

Histopathological Diagnosis:

Based on histological appearance, the diagnosis corresponds to an osteochondroma (exostosis).

Informed Consent:

In accordance with ethical guidelines, informed consent was obtained from the patient for the purpose of this case study and subsequent publication. Care was taken to ensure that the patient understood the nature of the case report, its potential benefits, and possible implications.

Privacy and Anonymity:

To protect the patient’s privacy, all identifying information has been removed or altered in the case report and any accompanying images. The patient’s anonymity has been preserved to the fullest extent.

We affirm that the ethical guidelines were thoroughly followed while preparing this case report and literature review, keeping the welfare of the patient and the integrity of the scientific community in mind.

## 3. Results

During the six-month follow-up, the patient reported remarkable improvement in her shoulder pain and weakness. She stated that the symptoms had completely resolved, indicating a successful outcome of the surgical intervention. Upon examination, no scapular winging was observed, indicating the restoration of normal scapular positioning. Imaging studies conducted at the follow-up visit confirmed the complete excision of the osteochondroma, affirming the efficacy of the surgical procedure.

The patient’s post-operative recovery involved a two-week period of immobilization in an elbow sling. This approach allowed for the passive range of motion of the shoulder joint while ensuring the necessary protection and support. The patient’s progress was carefully monitored by a physical therapist throughout this immobilization period. Following the immobilization phase, a tailored rehabilitation program was initiated, focusing on active assisted range of motion (AAROM) and active range of motion (AROM) exercises. These exercises were performed under the supervision and guidance of the physical therapist.

The rehabilitation program aimed to gradually restore the patient’s shoulder function, strength, and mobility. The physical therapist closely monitored the patient’s progress, adjusting the exercises and intensity as needed. The program emphasized a gradual increase in shoulder movements and resistance exercises to improve muscle strength and stability. This approach ensured a safe and effective recovery process, minimizing the risk of complications and promoting optimal healing.

The patient’s commitment to the rehabilitation program, coupled with the expertise of the physical therapist, contributed to her successful recovery. As a result, the patient regained the ability to engage in her usual activities without any restrictions. She expressed high satisfaction with the outcome of the surgery, indicating her contentment with the resolution of her symptoms and the restoration of her shoulder function.

## 4. Discussion

The rarity of scapular winging associated with osteochondromas, particularly in the superomedial angle of the scapula, underscores the need for increased awareness and understanding of this condition. The erosion of scapular bone caused by osteochondromas in this specific location can lead to a range of debilitating symptoms, including pain, weakness, and functional limitations [5].

The impact on a patient’s quality of life can be significant, as these symptoms can interfere with daily activities and restrict participation in sports or physical exercises. Therefore, early diagnosis and appropriate management are crucial to address these challenges and improve patient outcomes.

Scapular winging associated with osteochondromas is an exceptionally rare condition, and its occurrence in the superomedial angle of the scapula is even rarer. Osteochondromas in this specific location can lead to erosion of the scapular bone, resulting in notable symptoms such as pain, weakness, and functional limitations. Therefore, surgical excision is considered the preferred treatment approach for symptomatic osteochondromas in this context. However, in cases where the osteochondroma is asymptomatic, observation may be a suitable management option [6].

The manifestation of scapular winging with osteochondromas can significantly impact the patient’s quality of life. The associated pain, weakness, and functional restrictions can hinder daily activities and limit participation in sports or physical exercises. Thus, timely diagnosis and appropriate management are crucial to address these debilitating symptoms [7].

Surgical excision remains the treatment of choice for symptomatic cases of scapular osteochondromas. The selection of the surgical approach depends on factors such as the size and precise location of the osteochondroma. During the surgical procedure, it is imperative to meticulously dissect any adherent muscle or soft tissue to ensure complete removal of the osteochondroma, minimizing the risk of recurrence.

Postoperative rehabilitation plays a critical role in restoring shoulder and scapula function. A comprehensive physical therapy program is typically implemented to facilitate a gradual recovery and regain optimal range of motion and strength. The program may involve active-assisted and active range of motion exercises, guided by a physical therapist. Close follow-up is necessary to monitor the patient’s progress, assess the effectiveness of the treatment, and identify any potential signs of recurrence [8].

In the presented case, the patient experienced a successful outcome following surgical excision of the scapular osteochondroma. The complete resolution of symptoms and restoration of normal function, as reported by the patient, highlights the effectiveness of the chosen treatment approach. The patient’s ability to return to her usual activities without any restrictions and her high satisfaction with the surgical outcome further underscore the importance of early diagnosis and appropriate management of scapular winging associated with osteochondromas.

The clinical implications emanating from the presentation of this case study possess a dual significance. First, the case underscores the necessity of contemplating osteochondromas as a plausible etiology of scapular winging, with particular emphasis on the younger patient demographic wherein the incidence of osteochondromas is more prevalent. Second, the case delineates the pertinence of surgical intervention in instances where osteochondromas engender substantial symptomatic manifestations.

The salient points to be extracted from this case presentation encompass the following:

(1) In the clinical evaluation of scapular winging, particularly in younger patients, osteochondromas ought to be acknowledged as a potential causative factor;
(2) Osteochondromas engendering pronounced symptomatic repercussions may necessitate surgical intervention;
(3) The determination of a therapeutic approach to manage an osteochondroma should be individualized, taking into consideration various factors such as the severity of symptomatology, the dimensions and anatomical position of the tumor, and the patient’s age and degree of physical activity.

Scapular winging associated with osteochondromas is an infrequent yet impactful condition.

Optimal management entails surgical excision for symptomatic cases, while asymptomatic ones may warrant observation. Adherence to meticulous surgical approaches, postoperative physical therapy, and regular patient monitoring forms a holistic treatment plan.

Osteochondromas, including those occurring at the superomedial angle of the scapula, often present in a similar way across patients. These benign bone tumors typically develop during childhood or adolescence and can lead to skeletal deformities or complications related to surrounding soft tissue structures [4]. The clinical presentation often includes palpable mass, pain, limited range of motion, and mechanical symptoms such as snapping or popping. When occurring in the scapula, they can result in winging due to the proximity to nerves and soft tissue [9].

However, clinical presentations are generally similar across genders. It is worth mentioning that while the location of the osteochondroma on the superomedial angle of the scapula might be less common, the presentation and complications such as scapular winging can be seen in both genders [9].

The outcomes for patients with osteochondromas are generally good, with surgical excision often resolving symptoms. Young patients, in particular, tend to recover well and regain normal function, especially when the osteochondroma has been causing mechanical symptoms.

However, differences in outcomes can arise due to a variety of factors. For instance, the precise location and size of the tumor, the patient’s overall health and age, and the presence of any complications such as malignant transformation, which is rare but can occur.

The case of a young female patient with an osteochondroma at the superomedial scapula angle could therefore present similarities in clinical presentation to other cases of osteochondromas but may also have unique aspects due to the specific location of the tumor and the patient’s gender and age. The outcome for this patient is likely to be positive following surgical intervention, consistent with the general prognosis for osteochondromas.

The case accentuates the merit of prompt intervention, demonstrating substantial potential for symptomatic and functional improvements. Greater research and case documentation are vital to enriching our grasp of this condition, thereby refining diagnosis, treatment protocols, and overall patient care.

## 5. Conclusions

Osteochondromas occurring in the scapula are considered rare, and those specifically located in the superomedial angle of the scapula are even rarer. When present, these osteochondromas can give rise to various symptoms including scapular winging, weakness, and pain. The management of symptomatic cases typically involves surgical excision, which is considered the treatment of choice. However, due to the limited number of reported cases and the rarity of this condition, further research is needed to enhance our understanding of its pathophysiology and to establish optimal management strategies.

Scapular winging with osteochondromas is an uncommon condition that can significantly impact the affected individuals. The associated pain, weakness, and functional limitations can hinder daily activities and impair overall quality of life. In some cases, osteochondromas in the scapula may not cause any symptoms and can be managed through observation, while in symptomatic cases, surgical intervention is recommended.

The primary objective of surgical treatment is the complete removal of the osteochondroma along with any associated bone or soft tissue. This approach aims to alleviate symptoms, restore normal scapular and shoulder function, and prevent potential complications or recurrence. Postoperative physical therapy is often necessary to facilitate rehabilitation and optimize the recovery of shoulder and scapula function. Physical therapists work closely with patients to develop individualized rehabilitation programs that may include exercises to improve range of motion, strength, and functional abilities.

Despite the available literature on scapular winging with osteochondromas being limited, further research is essential to improve our knowledge and understanding of this rare condition. Areas of investigation may include the underlying pathophysiological mechanisms, risk factors, optimal surgical techniques, long-term outcomes, and potential complications. Collaborative efforts among researchers, clinicians, and experts in the field are necessary in order to gather more data, conduct comprehensive studies, and establish evidence-based guidelines for the management and treatment of scapular winging associated with osteochondromas.

In summary, osteochondromas occurring in the scapula, particularly in the superomedial angle, are rare. They can result in scapular winging, weakness, and pain, and surgical excision is generally recommended for symptomatic cases. However, due to the limited number of reported cases, further research is warranted to improve our understanding of the pathophysiology and optimal management strategies for this rare condition. By advancing our knowledge in this field, we can ultimately enhance patient care, optimize treatment outcomes, and improve the quality of life of individuals affected by scapular winging with osteochondromas.

## Data Availability

Not applicable.
